# Computer vision and machine learning for robust phenotyping in genome-wide studies

**DOI:** 10.1038/srep44048

**Published:** 2017-03-08

**Authors:** Jiaoping Zhang, Hsiang Sing Naik, Teshale Assefa, Soumik Sarkar, R. V. Chowda Reddy, Arti Singh, Baskar Ganapathysubramanian, Asheesh K. Singh

**Affiliations:** 1 Department of Agronomy, Iowa State University, Ames, IA, USA; 2 Department of Mechanical Engineering, Iowa State University, Ames, IA, USA

## Abstract

Traditional evaluation of crop biotic and abiotic stresses are time-consuming and labor-intensive limiting the ability to dissect the genetic basis of quantitative traits. A machine learning (ML)-enabled image-phenotyping pipeline for the genetic studies of abiotic stress iron deficiency chlorosis (IDC) of soybean is reported. IDC classification and severity for an association panel of 461 diverse plant-introduction accessions was evaluated using an end-to-end phenotyping workflow. The workflow consisted of a multi-stage procedure including: (1) optimized protocols for consistent image capture across plant canopies, (2) canopy identification and registration from cluttered backgrounds, (3) extraction of domain expert informed features from the processed images to accurately represent IDC expression, and (4) supervised ML-based classifiers that linked the automatically extracted features with expert-rating equivalent IDC scores. ML-generated phenotypic data were subsequently utilized for the genome-wide association study and genomic prediction. The results illustrate the reliability and advantage of ML-enabled image-phenotyping pipeline by identifying previously reported locus and a novel locus harboring a gene homolog involved in iron acquisition. This study demonstrates a promising path for integrating the phenotyping pipeline into genomic prediction, and provides a systematic framework enabling robust and quicker phenotyping through ground-based systems.

Iron (Fe) is an essential nutrient for a plant’s survival and propagation. It is involved in many important biological processes in plants including photosynthesis and respiration. Iron exists in soil in ferrous (Fe^2+^) and ferric (Fe^3+^) forms. Ferrous iron is soluble in soils but is readily oxidized by atmospheric oxygen^1^. At pH 7.4–8.5 or calcareous soils, ferric iron is predominant and has low solubility in the soil^2^. Plants utilize two strategies to take up Fe^3+^. Generally, graminaceous species secrete phytosiderophores (PS) and transport Fe^3+^ into the roots in an Fe-PS complex, and non-graminaceous plants reduce Fe^3+^ through membrane-bound reductases before being transported by a Fe^2+^ transporter^3^. Iron deficiency occurs when plants are unable to take up iron from the soil.

Iron deficiency chlorosis (IDC) is an important yield-limiting factor of soybeans [*Glycine max* (L.) Merr.] grown in the upper Midwestern United States (US) where calcareous soil is prevalent^4^. The typical soybean symptom of iron deficiency is interveinal chlorosis which is caused by a decrease in photosynthetic pigments in leaves due to a lack of iron^5^. Severe IDC damage, such as growth retardation and leaf necrosis, can lead to significant yield losses. The economic loss attributed to soybean IDC was estimated at 260 million dollars in 2012^6^ making it a commercially important trait for genetic study.

Previous studies suggested that soybean IDC resistance is controlled by a major-effect locus (*R*^2^ is at 25–80%) on chromosome 3 (Gm03) along with many loci with smaller effect across 10 of the 20 chromosomes^7^,^8^,^9^,^10^. Further introgression mapping of near isogenic lines of the major locus on Gm03 identified two soybean orthologs of the subgroup lb *basic helix-loop-helix (bHLH*) genes which are known to regulate the iron deficiency response genes in *Arabidopsis*^6^. A recent genome-wide association (GWA) study of IDC using advanced soybean breeding lines detected a group of loci harboring gene homologs involved in iron uptake and transport^11^, indicating a powerful tool of GWA study in dissecting genetic basis of complex trait. GWA analysis using a natural, diverse population has been repeatedly shown to be an efficient way to discover novel genetic variants of traits^12^,^13^,^14^,^15^, which is critical to create cultivars with robust and durable resistance to biotic or abiotic stresses. However, the natural genetic variants of IDC tolerance of the USDA soybean germplasm collection remain unexplored.

One major challenge of population genetics is the efficient and precise phenotyping of a large population with replicated tests. Current soybean IDC phenotyping is heavily based on visual assessment and SPAD measurement of chlorophyll concentration. Visual assessment is incapable of capturing small yet critical phenotypic variation and is plagued by the lack of intra-rater repeatability as well as inter-rater reliability^16^,^17^. Additionally, SPAD measurements are limited to a small fraction of the leaf, and are usually not statistically representative of the disease severity over the complete canopy. Furthermore, both methods are labor-intensive and time-consuming. These barriers in phenotyping have driven intense efforts by agricultural researchers and engineers to adapt newer technologies in field phenotyping. This has resulted in a wide variety of non-invasive and non-destructive methods of phenotyping such as remote sensing^18^, handheld sensors^19^, and more recently, digital imaging^20^,^21^. With the advances in data analytics, machine learning (ML) has been shown to be critical for image phenotyping of stress-related traits in plants^22^.

The current state-of-art in sensor platforms, computing hardware and automation, as well as recent advances in machine learning principles make the phenotyping workflow that seamlessly integrates image phenotyping and ML-based features extraction a promising and potentially transformative approach to accelerate genetic gain. Motivated by this, we report GWA and genomic prediction (GP) analyses of IDC tolerance in soybean using a large diverse germplasm panel through an ML-based image-phenotyping pipeline. The workflow for phenotyping (image capture → data storage and curation → trait extraction → ML-enabled classification and quantification) was designed and efficiently executed to generate IDC score and severity. The reliability and advantages of the phenotyping pipeline are further illustrated via GWA analyses. Modeling the major genetic variant and/or a surrogate trait as fixed effects is demonstrated to enhance GP when compared to models with all factors as random.

## Results

### Image-acquisition, image-processing and ML-enabled phenotyping

A total of 5,916 red-green-blue (RGB) images of individual soybean plots was taken at the same time as field visual rating (FVR) by flowing a standard digital imaging protocol to obtain consistent images of individual plant canopies (Box 1). Stages of image processing, quality control and domain expert informed feature extraction are described in the methods section. Finally, 4,366 images were used for further study. The extracted features are mapped to a standard visual IDC ratings scale through a ML based hierarchical classification strategy. This choice was motivated by the presence of unbalanced data (Table S1). The preponderance of IDC score 1 made designing classifiers with multiple classes problematic. A multi-class Support Vector Machine (SVM) model proved adept at categorizing the observations into three groups (low, medium and high IDC susceptibility groups which was based on expert elicitation of information about IDC susceptibility); an individual SVM was then deployed on each susceptibility group to identify the corresponding IDC classes. Accuracy and average per-class accuracy were 99.4% and 95.9%, respectively, using this hierarchical SVM + SVM classifier. The decision boundaries of the SVM + SVM classifier were presented in Fig. S1. Except FVR of 3 (Sensitivity = 0.8), sensitivity and specificity for all other rating classes were 1. As an additional check of the framework, we conducted a separate experiment to acquire 124 images of PI lines that were not part of the association panel. The images of the separate experiment were collected by following the same protocol as described above. The accuracy and average per-class accuracy were 96.0% and 90.6%, respectively. The severity function was then designed to associate a single scalar value to each image/genotype (Table S2).

### Identification of the major QTL linked in repulsion for IDC

**Figure 1:**
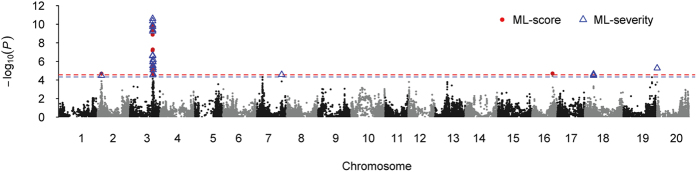
Manhattan plots of genome-wide association analysis with machine learning IDC score (ML-score) and severity (ML-severity) in soybean. Negative log10-transformed *P* values from a genome-wide scan for IDC resistance by using a mixed linear model are plotted against positions on each of the 20 chromosomes. The red and blue dash lines indicate the significance level of FDR = 0.05 for ML-score and ML-severity, respectively. The significant SNP were highlighted in red for ML-score and blue for ML-severity.

Genome-wide association analyses were conducted using both the ML-classification based IDC score (also called ML-score) and the severity (also called ML-severity) of the V5-V6 growth stage. GWA analyses identified 19 and 27 single nucleotide polymorphisms (SNPs) associated with the ML-score and ML-severity, respectively (Fig. 1). Among them, 17 SNPs were common and tagged to a region of 847 kb on Gm03. All significant SNPs were finally clustered into seven QTL associated with soybean IDC resistance (Table 1). Three loci, *ss715583665* (Gm02), *ss715585452* (or *ss715585486*) on Gm03, and *ss715585473* (Gm03), were associated with both traits. The *ss715585452* alone contributed 8.0–9.1% of the total phenotypic variation, representing the largest effect locus in the present study. Notably, *ss715585452* and *ss715585473* were 173 kb apart on Gm03 (Table1). Further investigation indicated that these two loci were in coupling phase in 62% (288) of the association panel and 37% (171) were in favorable allelic combination (Fig. S2).

### Allelic study in the soybean cultivar panel

**Figure 2:**
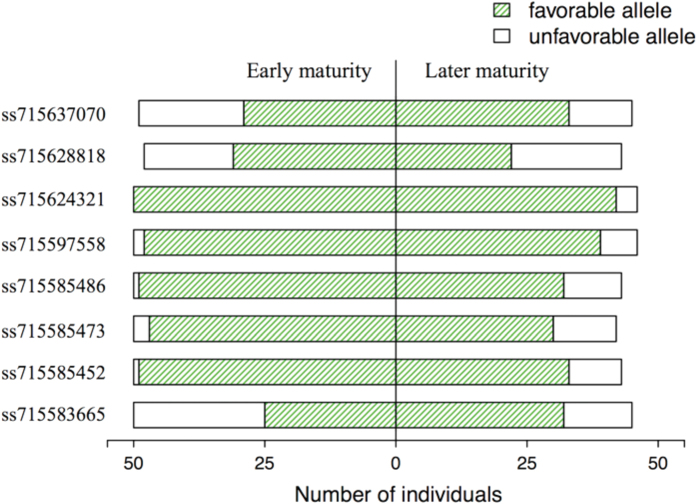
Allelic frequency of all the QTL associated with IDC resistance in a sample of 96 elite soybean cultivars.

To understand the allelic status of the identified loci in soybean cultivars, especially the two linked QTL, *ss715585452* and *ss715585473* on Gm03, 96 elite cultivars that were released in the US and Canada mainly during the 1990s and described in a previous study^23^ were investigated. At least 50 of the 96 cultivars belonged to geographical regions where IDC is present and in earlier maturity groups adapted to the north-central states of the US and Canada (Table S3). Among the total population, 79% (76/96) were in coupling linkage with favorable alleles at the two linked QTL. This number increased to 92% (46/50) when only the early-maturing cultivars were considered (Fig. 2). The above results indicated that, similar to the association panel, the coupling linkage with resistant alleles of the QTL on Gm03 was prevalent in the elite cultivars under testing, especially for those adapted to the north-central region in the US.

### Candidate genes

**Figure 3:**
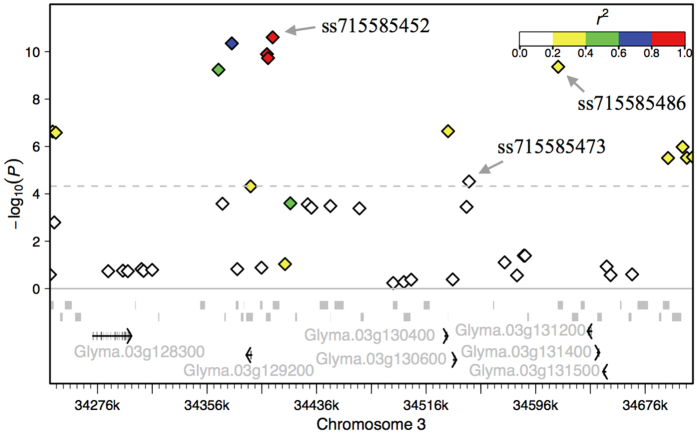
The chromosomal region for loci associated with IDC tolerance on Gm03. Negative log_10_-transformed *P* values from the association mapping for ML-severity using mixed linear model are plotted on the vertical axis. The x-axis is the physical position on the chromosome. The gray dash line indicates the significance level at FDR = 0.05 (P = 4.0 × 10^−5^). The diamonds represent SNPs. The three lead SNPs of loci associated with ML-score and/or ML-severity are indicated by arrows. The color of each SNP indicates the linkage disequilibrium *r*^2^ value with the peak SNP, *ss715585452*, as shown in the color intensity index on top-right. All of the transcripts are given as indicated by the gray segments. Seven candidate genes were highlighted in black, and gene names were given.

The dense markers at the detected chromosomal regions encouraged a further search of candidate genes involved in iron metabolism in soybean. Within the 847 kb region on Gm03 associated with both ML-score and ML-severity, seven candidates were identified at the proximity of the peak SNPs (Fig. 3). A cluster of three 2OG-Fe(II) oxygenase superfamily genes, *Glyma.03g131200, Glyma.03g131400* and *Glyma.03g131500*, were 21.4 kb apart from the peak SNP, *ss715585486,* associated with ML-score. *Glyma.03g129200,* encoding a cytochrome P450 subfamily protein, and *Glyma.03g128300,* encoding a ferredoxin-dependent glutamate synthase (GLUS), were located at 15.4 kb and 103.1 kb upstream of *ss715585452*, the lead SNP associated with ML-severity. Two neighboring *bHLH* transcription factor genes, *Glyma.03g130400* and *Glyma.03g130600,* resided 9.2 kb away from *ss715585473*. These two genes were previously reported to be responsive to iron deficiency in soybean^6^. The candidate gene for the locus *ss715628818* on Gm18, specifically associated with ML-severity, was also identified. *Glyma.18g111000,* encoding another 2OG-Fe(II) oxygenase superfamily protein, was found at 41.4 kb downstream of the lead SNP.

### Genomic predictions

**Figure 4:**
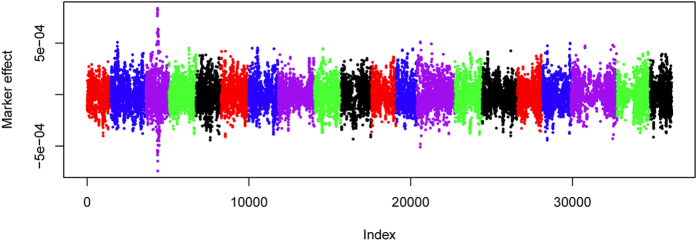
Estimates of the marker effect in genomic prediction with rrBLUP. The x-axis indicates the position index of 35,683 SNP in the entire soybean genome. Chromosomes are distinguished by colors. From left to right is chromosome 1 to chromosome 20. The y-axis indicates the SNP effect.

When all of the SNPs were used as random effects, the prediction accuracy was 0.44 for the ML-score and 0.37 for the ML-severity (Table 2). Consistent with GWA analyses results, two peaks with effects in the opposite direction were observed at the chromosomal region on Gm03 where the two QTL were identified (Fig. 4). Specifying the major locus, *ss715585452*, as a fixed effect and the genome-wide markers as random effects increased the prediction accuracy to 0.51 and 0.46 for ML-score and ML-severity, respectively. When the SPAD data were treated as a fixed effect and the genome-wide markers as random effects, the prediction accuracy further increased to 0.55 and 0.51 for ML-score and ML-severity. The prediction accuracy reached the highest at 0.58 and 0.56 when the major QTL and SPAD values were considered as fixed covariates (Table 2).

## Discussion

Machine learning automates the process of pattern and trend extraction from the images and helps the identification, classification and quantification of the objects^22^. In the present study, an ML pipeline was adopted to quantify soybean IDC symptoms by ranked ML-score and continuous ML-severity using digital images of the plant canopy. The digital imaging-based disease qualification workflow is built on a tight integration of (a) field-based digital imaging of the canopy, (b) automated pre-processing of the collected images to improve the signal-to-noise ratio (removing background and other spurious features), (c) expert knowledge-based feature extraction of IDC symptoms, and (d) ML-based classification and severity function generation. The complete workflow is fully automated, thus representing a seamless integration of several recent advances in imaging and ML. The high prediction accuracy (>0.95) of the ML-score provides appealing prospects for the substitution of human visual rating with digital image based phenotyping of traits amenable to description in a domain knowledge context so that features can be extracted from images.

The GWA analyses results further validate the viability of the image phenotyping of IDC in soybean. The major QTL, *ss715585452* (MAF = 0.31) on Gm03, identified for both ML-score and ML-severity, has been repeatedly reported in previously studies^6^,^8^,^9^,^10^ and overlaps with the region where the IDC-related genes had been finely mapped^6^. In the present study, however, a new QTL (*ss715585473*) tightly linked to *ss715585452* was identified, indicating the complex genetic architecture under this chromosomal region. The identification of the new QTL likely benefited from the diverse panel and the high-dense marker employed in the current study. The low LD (*r*^2^ = 0.11) between the two leading SNPs further suggests the independence of the loci. The existence of two QTL at this region was further confirmed by the drastic change of the loci effects as identified in GP. Because of the tight linkage of the QTL, it is difficult to distinguish from each other through linkage mapping. Consequently, the effect of the known major QTL in previous studies may be overestimated due to the prevalent phase of coupling linkage in both the diverse panel and the elite cultivars.

Consistent with the complexity of the genetic architecture of the region on Gm03 as revealed above, multiple genes related to iron metabolism were identified within this region. Two of them, *Glyma.03g130400* and *Glyma.03g130600* (also known as *Glyma03g28610* and *Glyma03g28630,* respectively) encode *bHLH* transcript factors. Their expressions were root specific and iron stress inducible^6^. Another candidate gene, *Glyma.03g128300*, putatively encodes a ferredoxin-dependent GLUS and primarily expresses in leaf and stem. Loss function of its homolog in *Arabidopsis* results in leaf chlorosis (http://www.arabidopsis.org). The fourth candidate gene is *Glyma.03g129200*. It is highly expressed in root hair and is proposed to encode a cytochrome P450 protein containing a domain that interacts selectively and non-covalently with iron ions (https://phytozome.net).

The major QTL on Gm03 was not detected in previous GWA studies for IDC where 132 and 138 advanced soybean breeding lines, developed by public and private breeding programs adapted to the north-central states of the US, were used^11^,^24^. The identification of the major QTL in these studies may have been prevented due to rare allele because elite by elite crosses are common for soybean cultivar development, and the resistance allele of the QTL in older elite cultivars was likely fixed as illustrated in this study, especially for those grown in north-central US. However, the persistence of large yield losses caused by IDC suggests the necessity to explore novel genetic variants for soybean IDC tolerance improvement.

Additionally, it is important to emphasize the advantages of the image phenotyping pipelines present in this study. On one hand, automated image processing with ML-based algorithms is shown to provide a fast, efficient way to obtain abiotic stress evaluation. Here the output is the IDC scores, which is a discrete and finite number of classes (five in this case). It is subsequently used for GWA studies and GP. On the other hand, an alternative severity function is introduced that maps an image to a continuous severity index. This continuous mapping encodes both intra- and inter-class variation as opposed to simply the inter-class variation encoded in the IDC scores. Our results suggest that GWA studies performed on such continuous functions (ML-severity) provide a richer set of loci than the conventional class based descriptors (ML-score).

Two novel QTL, *ss715628818* (Gm18) and *ss715637070* (Gm20), were identified via GWA for ML-severity but not ML-score. One of them harbors the candidate gene *Glyma.18g111000*, which encodes a 2OG-Fe(II) oxygenase superfamily protein. The *Arabidopsis* homolog AtF6′H1 (70.9% similarity) catalyzes the conversion of feruloyl-CoA to 6′hydrooxy-feruloyl-CoA. It is the key step to redirect the flow of carbon toward the production of phenolics by preventing the entrance of feruloyl-CoA into lignin or suberin synthesis under iron deficiency conditions^25^. This redirection of carbon flow is critical for the plant to produce and secrete phenolic compounds and acquire iron from sources with low bioavailability such as the high pH soil that leads to IDC. Recent studies revealed that AtF6′H1 is essential for the tolerance of *Arabidopsis* to high pH-induced iron deficiency^26^.

It is desirable to integrate image-phenotyping and ML components into the GP pipeline to maximize genetic gain. GP estimates the trait breeding value using genome-wide markers instead of a subgroup of trait-associated markers^27^. However, simulation study suggested that there is a gain to model a major QTL (*R*^2^ > 10% of total genotypic variance) as a fixed versus random effect in multiple cycles of GP^28^. In the present study, the prediction accuracies were 0.51 and 0.46 for ML-score and ML-severity by specifying *ss715585452 (R*^2^ > 10% in the context of general linear model) as a fixed effect, which were 7% and 9% higher than treatment as a random effect. A previous study indicated that involving phenotypic information of the target trait-related trait in the genomic prediction model had the potential to improve the prediction accuracy^29^. SPAD measurement is based on the leaf chlorophyll concentration, which is highly correlated with IDC performance in soybean. In this study, involving SPAD as a fixed effect further increased the prediction accuracies, implying that the prediction accuracy of a trait based on marker information can be improved with the data from an additional trait. Considering the intermediate heritability of the trait^7^,^10^,^30^, the prediction accuracies achieved in this study were promising and indicated the great potential of using GP in prediction of soybean IDC resistance.

## Conclusions

The present study leveraged a ML-enabled image phenotyping to GWA and GP analyses for IDC in soybean. The results demonstrate the reliability of this phenotyping pipeline for genetic study and emphasize its advantage in capturing the minor phenotypic variance of the trait. This study also illustrates the complexity of the genetic basis of soybean IDC resistance and stresses the necessity of new resistant resources for IDC improvement. The significant improvement of the prediction accuracy shows the great potential of specifying the major QTL and/or a surrogate trait as fixed covariants in GP for complex traits. The ML-enabled image phenotyping pipeline presented in this study can be extended to other traits and crops, and the integration of this pipeline into the ground- or aerial-based systems holds significant promise for accelerating genetic gain of cultivar development breeding.

## Methods

### Plant material and field phenotyping

A total of 478 soybean lines including 473 plant introduction (PI) lines, three maturity checks and the IDC resistant (Clark) and susceptible (Iso-clark) checks were included in this study. The PI lines were acquired from the USDA soybean germplasm collection, and were maturity group (MG) I (31%), II (36%) and III (33%). They originated from 27 countries across the world, and the top three origin countries were China (59%), Japan (17%) and Korea (8%). These PIs are a subset of the core collection of the USDA soybean germplasm and are broadly representative of the diversity of the lines within MG I-III^31^. The whole panel was planted near Ames, IA, in 2015, where soybean IDC had been present in previous years, in a randomized complete block design with four replications. Within each replication, all checks were repeated four times resulting in a total 493 plots/rep. The plants were planted in hill plots of five seeds per plot at an inter- and intra-row distance of 0.31 m and 0.61 m, respectively.

FVRs and digital images of the plant canopies were collected at the second to third trifoliate (V2-V3) and the fifth to sixth trifoliate (V5-V6) growth stages and two weeks later as well. A scale of 1 to 5 was used for FVRs as described previously^8^. Briefly, 1 indicates no chlorosis, and plants were normal green; 2 indicates plants with modest yellowing of the upper leaves; 3 indicates plants with interveinal chlorosis in the upper leaves but no stunting; 4 indicates plants with interveinal chlorosis and stunted growth; and 5 indicates that plants show severe chlorosis, stunted growth, and necrosis in the youngest leaves and growing points. The average yield loss was 20% for each increase in IDC score^32^. However, the SPAD measurements of the upper leaves and the soil samples were taken at the V2-V3 and the V5-V6 stages only. The SPAD value of each plot was measured using the SPAD chlorophyll meter as described in a previous study^33^. At each stage, eight soil samples were randomly collected from each replication using a JMC soil probe (Clements Associates, Inc., Newton, IA) at a depth of 16 cm and were mixed together as one sample for test in the Soil and Plant Analysis Laboratory, Iowa State University. The soil pH values ranged from 7.80–7.95 and 7.75–7.85 at the V2-V3 and V5-V6 growth stages, respectively. The canopy images, SPAD measurements and the soil samples were taken at the same time as FVRs.

### Imaging Acquisition

Images were taken using a Canon EOS REBEL T5i camera with the Scene Intelligent Auto model. All images were stored in RAW image quality with a resolution of 5184 × 3456 (18 M). For consistent illumination, the flash function was kept off, and an umbrella was always used to shade the area under the camera view. A picture of the X-Rite Color Checker Color Rendition Chart was taken at the beginning of imaging operations and every 20 min thereafter or whenever the light condition changed (e.g., cloud cover) to calibrate the illumination of plant canopy images collected at that moment. When taking pictures, the whole canopy was fit in the field of view of the camera. Whenever possible, weeds and other plant residuals, connected to the plant canopy in the view of camera, were removed for easy image processing and enhanced efficiency of subsequent image processing. A detailed imaging protocol is shown in Box 1.

### Image Preprocessing

#### Segmentation

Each image was converted from native RGB to hue-saturation-value format to efficiently segment the foreground (the plant) from the background. The background of an image (soil) contains more gray pixels than the foreground (plant) does and lacks in green and yellow hue values; therefore, most of the background was removed by excluding pixels that had a saturation value below a predefined threshold and hue values outside of a predefined range. The saturation threshold value was obtained by identifying the saturation values of the background in 148 diverse images. This result provided consistent segmentation across fields. The hue range was simply obtained from the hue color wheel, removing pixels that were neither green nor brown.

#### Outlier removal and cleanup

**Figure 5:**
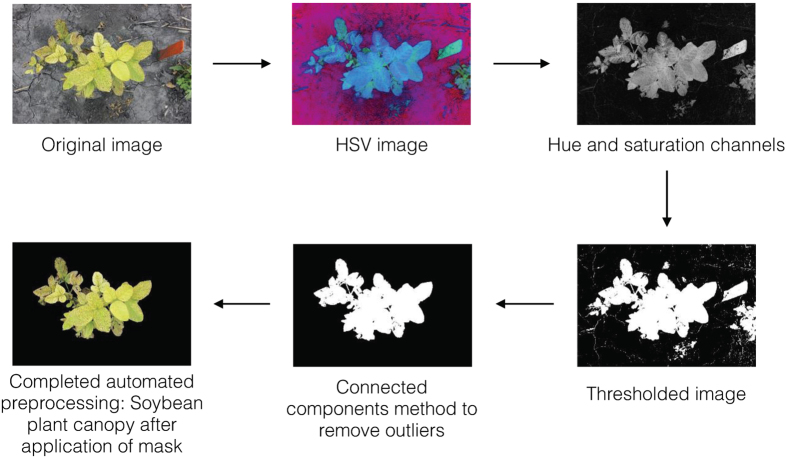
Image preprocessing overview on iron deficiency chlorosis-impacted plant canopies in soybean. HSV, hue-saturation-value.

Once segmentation was done, the connected components method was used on the processed image to remove spurious outliers and noise from the image (e.g., plant debris on soil). This was accomplished by identifying clusters of pixels that connected to one another, labeling them, and identifying the largest connected component (i.e., plants in a plot). Cleaning was done by removing any other connected components that contained fewer pixels than the largest connected component. Then, a mask of the isolated plant was applied to the original RGB image in order to display the isolated plant in color. In contrast to other commonly used thresholding methods^28^, no significant pixel loss was observed. The preprocessing sequence is illustrated in Fig. 5.

All analysis was performed using the image processing toolbox and image processing functionality available in Matlab. Specific functions included segmentation, color map conversion and the connected components algorithms. All analysis routines are packaged into Matlab.m files and are available upon request.

### Feature extraction based on expert elicitation

**Figure 6:**
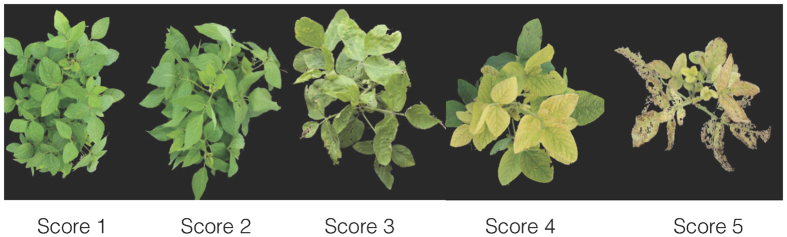
Field visual rating examples of soybean canopies with iron deficiency chlorosis scores from 1 to 5.

**Figure 7:**
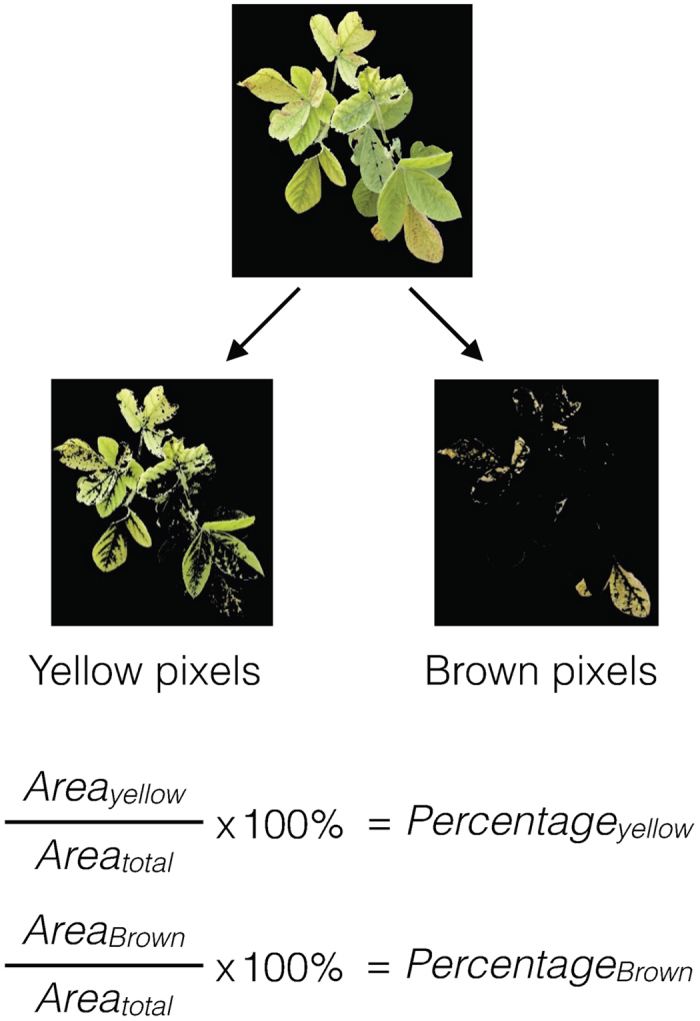
Extraction of yellow and brown pixel from the images of soybean canopies impacted by IDC and subsequent calculation of yellow and brown percentages for each image.

The FVR is based on yellowing and necrosis (Fig. 6); therefore, color changes (green to yellow to brown) were used as the trait signature for the measure of severity. Hue values were used to identify pixels that were yellow and brown, and the extent of discoloration was represented as the percentage of the yellow and brown areas of the canopy (Fig. 7).

### Classification

Each image was represented by a quantitative measure of the spread of discoloration [i.e., fraction of the canopy that was yellow (exhibiting chlorosis), yellow% (Y%), and fraction of the canopy that was brown (exhibiting necrosis), brown% (B%)]. Hierarchical classification, which is a supervised learning approach, was used to map these quantitative visual measures to the visually rated IDC severity to construct an automated IDC classifier. FVR was the label used for classification, and the vector (B%, Y%), representing the fraction of canopy area exhibiting necrosis and chlorosis, served as the input. The dataset (4,366 images) was randomly divided into two sets: the training dataset (75%) and the testing dataset (25%). We calculated three measures of classifier accuracy: (a) overall accuracy, (b) per-class accuracy, and (c) average per-class accuracy (Table S4).

**Figure 8:**
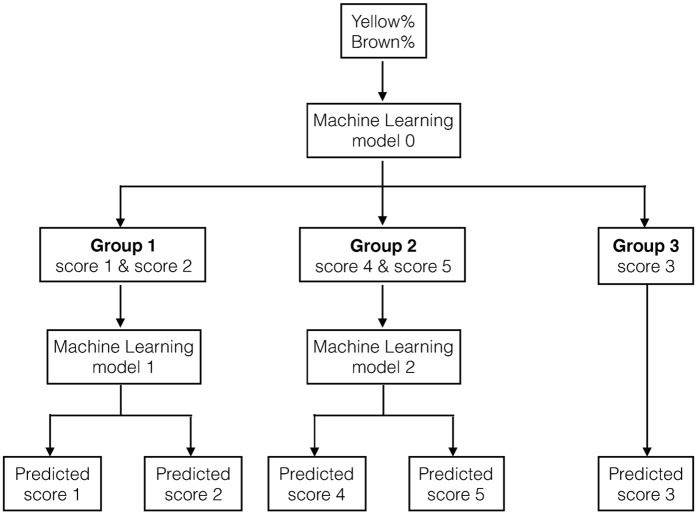
Flowchart of the hierarchical classifier that was built to map the extracted features (Yellow %, Brown %) to the IDC score. Box 1. Always take a picture of the X-Rite Color Checker Color Rendition Chart first. (**a**) Ensure that the lighting does not change after taking a picture of the chart. (**b**) Should the lighting change, take a picture of the chart again. (**c**) Do not touch the colored squares on the chart. 2. Ensure that no weeds, other plants, or large objects (e.g., shoes, paper, and so forth) merge with the plant(s) canopy in the image. 3. If taking pictures of greenhouse plants, ensure that the background of the image is one flat color; black is preferred. Use a black cloth to cover the background. 4. Take pictures of the entire plant(s) canopy. 5. Ensure that light is not reflected by the leaves; in this case, leaves appear white. Try not to use flash. If using a flash is essential, use a diffuser on the flash to have even lighting. 6. Always take images from a top-down view.

Several classifiers including random forests, decision trees, support vector machines were tested. A hierarchical multilevel linear support vector machine (SVM) was the best classifier. In the first level, an SVM-based classifier separates the data into low, medium, and high susceptibility groups based on their yellow and brown percentages. In the second level, SVM is again deployed to further classify the susceptibility groups into score 1 or 2 (for the low susceptibility group), and score 4 or 5 (for the high susceptibility group). A flowchart of the hierarchical classification process is illustrated in Fig. 8.

Following this, a quantitative severity function is constructed that maps the vector (B%, Y%) to a single number between 0 and 100. Here, 0 indicates no IDC symptoms, while 100 indicates substantial necrosis. A lower IDC score has correspondingly low values of the severity function while high IDC scores have high values of the severity function. Within an IDC rating class, specimens with higher necrosis (and chlorosis) have higher values of severity function. Severity mapping was formulated as a weighted linear combination of the components of the vector (B%, Y%) because 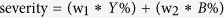. The weights (w1 and w2) were obtained by an optimization routine that minimizes the misclassification error (Table S2).

### Genotyping and quality control

A total 465 of the 473 PIs was genotyped using the Illumina Infinium SoySNP50K BeadChip in a previous study^23^, and the SNP dataset was downloaded from SoyBase (http://soybase.org/). A total of 42,509 SNPs were identified. Among them, 60 unanchored SNPs were excluded from further analysis resulting in a dataset with a 0.5% missing rate. SNPs with a missing rate > 10% were ruled out. The remaining missing data were imputed using BEAGLE version 3.3.1 with default parameter settings^34^,^35^. SNPs with a minor allele frequency (MAF) <5% were excluded from analysis as well. Finally, a total of 36,139 SNPs were used for GWA analyses and GP. The genotypic data of the 96 elite soybean cultivars has been reported in previous articles^23^.

### Genome-wide association analysis

The V5-V6 growth stage and the time point 3 (two weeks post V5-V6) had similar variation of FVR (score 1–5) but larger than that of the V2-V3 stage (score 1–4). However, soil pH values and SPAD measurements were collected at V5-V6 but not time point 3. Consequently, the ML-score and ML-severity of V5-V6 growth stage were further applied to genome-wide studies. Due to phenotypic data missing, 461 of the 465 genotyped PIs were involved in further study. The best linear unbiased predictions (BLUPs) of the trait breeding value of each line over four replications were calculated using the R package, lme4^36^,^37^, and were then used for GWA studies. The GWA analyses were implemented using the Genomic Association and Prediction Integrated Tool (GAPIT)^38^,^39^.

The mixed linear model (MLM) equation was:


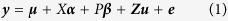


where y is an *N* × 1 vector of the IDC trait BLUPs, *N* is the number of lines, *μ* is the overall mean, *X* is the marker genotypic matrix relating the individuals to the fixed marker effects *α, P* is the incidence matrix relating the individuals to the fixed principal component effects *β* based on the entire SNP dataset. In the present study, however, the model fitness test using Bayesian information criteria suggested no PC to be involved into the association analysis. *Z* is the incidence matrix relating the individuals to the random group effects *u*. The random group effect, *u*, follows a multivariate normal distribution with a mean of 0 and a variance-covariance matrix of 2*KV*_*g*_, where *K* is the kinship matrix using the entire SNP dataset, and *V*_*g*_ is the genetic variance component. The random error term, *e,* follows a multivariate normal distribution with a mean of 0 and the variance-covariance matrix, *IV*_*e*_, where *I* is the identity matrix, and *V*_*e*_ is the error variance component. The significance threshold for SNP trait associations was determined by a false discovery rate (FDR) <0.05. The Bayesian Information Criteria model fitness test suggested that no principal component should be included in the MLM. To determine the trait-associated loci, all significant SNPs located in close physical proximity were clumped at pairwise linkage disequilibrium (LD) values of *r*^2^ > 0.20. Only the strongest trait-associated SNP (or peak SNP) within each LD block was kept.

### Genome-wide prediction

Genomic predictions for ML-score and ML-severity were conducted by modeling the GWA-identified loci and SPAD measurements as having fixed effects and the genome-wide markers as having random effects with the ridge regression best linear unbiased prediction (RR-BLUP) approach. The RR-BLUP was performed using the rrBLUP package in the R programing language as described by Endelman (2011)^40^:


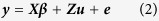


where *y* is a vector of the IDC trait BLUPs, *X* is a design matrix for the fixed effects *β, Z* is the marker genotypic matrix for the random marker effects (Var[*u*] = 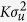, and *K* is the identity matrix in the present study), and *e* is the residual error with Var[*e*] = 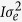. The genomic-predicted values of the traits were accessed through the ten-fold cross validation approach as described previously^41^. The prediction accuracy was calculated as the Pearson correlation (*r*) between the IDC trait BLUPs and the genomic-predicted values.

## Additional Information

**How to cite this article**: Zhang, J. *et al*. Computer vision and machine learning for robust phenotyping in genome-wide studies. *Sci. Rep.* **7**, 44048; doi: 10.1038/srep44048 (2017).

**Publisher's note:** Springer Nature remains neutral with regard to jurisdictional claims in published maps and institutional affiliations.

## Supplementary Material

Supplementary Tables and Figures

## Figures and Tables

**Table 1:** SNP loci significantly associated with ML IDC score and severity.

Trait	SNP	Chr	Position	MAF	FDR	Allelic effect	R^2^ (%)	Candidate gene	Annotation
ML-score	*ss715583665*	2	5855107	0.22	0.04	0.10	4.4		
	*ss715585473*	3	34547382	0.43	0.04	−0.08	3.5	*Glyma.03g130400 Glyma.03g130600*	bHLH^6^ bHLH^6^
	ss715585486*	3	34612476	0.23	0.00	0.15	8.0	*Glyma.03g131200 Glyma.03g131400 Glyma.03g131500*	2-oxoglutarate -Fe(II) oxygenase superfamily
	*ss715624321*	16	30708368	0.11	0.04	0.12	3.5		
ML-severity	*ss715583665*	2	5855107	0.22	0.04	0.97	3.4		
	ss715585452*	3	34403919	0.31	0.00	1.43	9.1	*Glyma.03g128300 Glyma.03g129200*	Ferredoxin-dependent GLUS Cytochrome P450
	*ss715585473*	3	34547382	0.43	0.04	−0.77	3.4		
	*ss715597558*	7	37177741	0.20	0.04	0.90	3.5		
	*ss715628818*	18	13099563	0.37	0.04	0.87	3.5	*Glyma.18g111000*	AtF6′H1^25^,^26^
	*ss715637070*	20	248491	0.07	0.01	1.47	4.1		

^*^SNPs are in LD with *r*^2^ > 0.2.

**Table 2:** Genomic prediction accuracies of different models developed for iron deficiency chlorosis in soybean using an association mapping panel.

Model	RR-BLUP	RR-BLUP with the major QTL and SPAD values as fixed effects ss715585452	SPAD	SPAD + ss715585452
ML-score	0.44	0.51	0.55	0.58
ML-severity	0.37	0.46	0.51	0.56
