# Supplementary material for: Computer vision and machine learning for robust phenotyping in genome-wide studies
Source: Sci Rep. 2017 Mar 8;7:44048. doi: 10.1038/srep44048 (PMC5358742; doi:10.1038/srep44048)
Supplement: Supplementary Tables and Figures [file srep44048-s1.pdf]

Supplementary information *for manuscript* '**Computer vision and machine learning for robust phenotyping in genome-wide studies**'

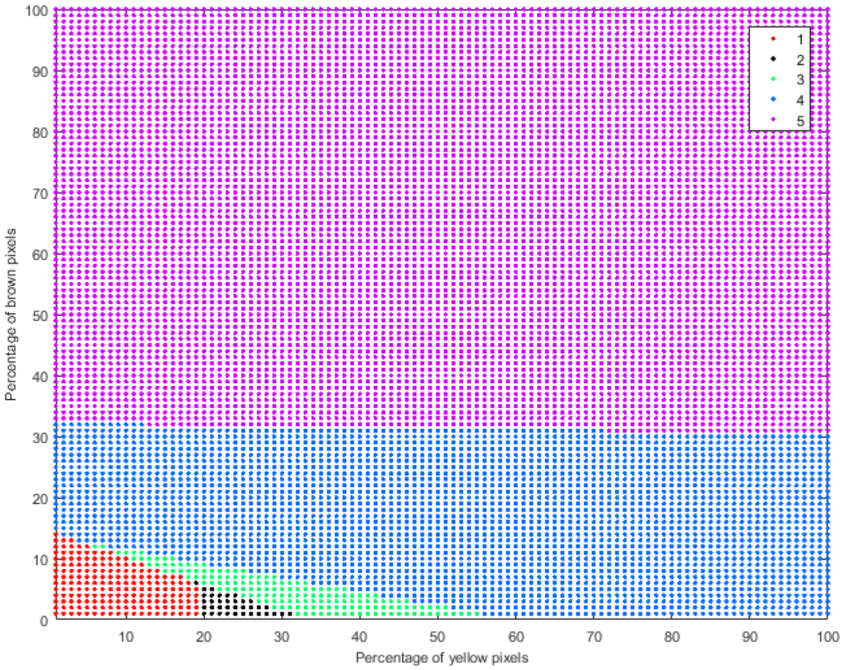

Figure S1. Soybean iron deficiency chlorosis classification decision boundaries of the support vector machine model based on the Yellow% and Brown% of canopy images.

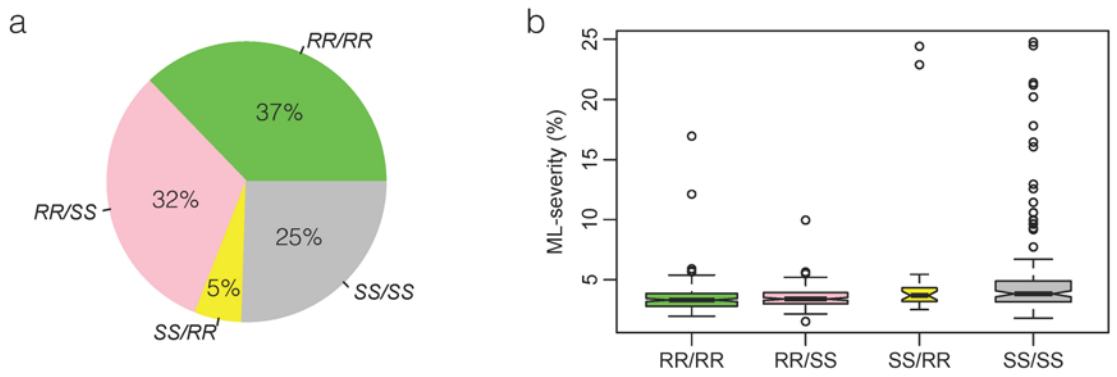

Figure S2. Frequency of genotype combinations of *ss715585452* and *ss715585473* and the phenotypic difference between the combinations in the 461 germplasm lines: **a**, a pie chart showing the proportion of each genotype combination of the two loci highlighted in colors, and **b**, a boxplot showing the difference of genotypic value (over four replications) of ML-severity among four genotype combinations presented in a format of *ss715585452*/*ss715585473*. RR indicates the resistant homozygous genotype; SS indicates the susceptible homozygous genotype. Two lines heterozygous at the *ss715585452* locus were excluded from the analysis.

Table S1. The class distribution of field visual rating (FVR) of the 4,366 iron deficiency chlorosis canopy images.

FVR	Number of Observations
1	4210
2	35
3	27
4	77
5	17

Table S2. Functions for the development of ML-severity of iron deficiency chlorosis in soybean.

Algorithm†	Method‡	Resubstitution Errors (%)
HM	1	3.09
	2	3.87
	3	9.75
LDA	1	1.16*
	2	1.08
	3	3.02
QDA	1	1.16
	2	0.40
	3	4.03

HM: Hierarchical Model, LDA: Linear Discriminant Analysis, QDA: Quadratic Discriminant Analysis

† These 3 algorithms were used because they performed the best in the task of classifying features to rating and did not require hyperparameter tuning (e. g. k in kNN).

‡ Method 1: $\text{Severity} = (w_1 * Y\%) + (w_2 * B\%)$ (1)

Method 2: $\text{Severity} = (w_1 \hat{} Y\%) + (w_2 \hat{} B\%)$ (2)

Method 3: $\text{Severity} = (w_1 * Y\%) * (w_2 * B\%)$ (3)

Method 2 provides the lowest resubstitution errors for both LDA and QDA but fails in instances where small changes in Y% affect the overall severity rating more than changes in B%. At this point, it conflicts with what is known--that plants that experience necrosis have considerably more symptom severity.

* The algorithm that was finally used for the ML-severity calculation.

Description:

The weights for this combination were obtained from an optimization routine called the genetic algorithm (GA). Weights that minimize the resubstitution error were determined based on classifying each severity to their respective ML IDC ratings. In other words, instead of having Y% and B% as predictors for the classification problem, the severity rating is utilized as the input, and a misclassification error is computed after evaluating the classification model using similar methods discussed in the classification section. The GA then iterates and produces weights that result in the minimal resubstitution error. Then, the resulting severity rating, with optimum weights, is normalized to provide a more intuitive scale of 0 - 100%.

Table S3. List of 50 cultivars from the 96 elite soybean cultivars panel used in the allelic study.

Item No.	Plant ID†	Maturity	Region adapt
1	AC_2001		Ottawa, Ontario, CA
2	AC_Albatros		Ottawa, Ontario, CA
3	AC_Brant		Ottawa, Ontario, CA
4	AC_Bravor		Ottawa, Ontario, CA
5	AC_Cormoran		Ottawa, Ontario, CA
6	AC_Harmony		Ottawa, Ontario, CA
7	AC_Orford		Ottawa, Ontario, CA
8	Accord		Ottawa, Ontario, CA
9	Alpha	I	
10	Apollo	II	
11	AYR		Ottawa, Ontario, CA
12	Barnes	0	
13	Blackjack_21		Ottawa, Ontario, CA
14	Brock	I	
15	Colfax	II	
16	Conrad_94	II	
17	Daksoy	0	
18	Dwight	II	
19	Faribault	I	
20	Felix	I	
21	Freeborn	I	
22	Glacier	0	
23	Granite	I	
24	Hendricks	0	
25	IA1008	II	
26	IA2007		Iowa, US
27	IA2022		Iowa, US
28	IA2050		Iowa, US
29	IA3003		Iowa, US
30	IA3004		Iowa, US
31	IA3005		Iowa, US
32	IA3010		Iowa, US
33	LN89_3264	II	
34	Medallion		Ottawa, Ontario, CA
35	MN0901	0	
36	MN1301	I	
37	OAC_Salem		Ottawa, Ontario, CA

38	OAC_Shire	I	
39	OAC_Stratford		Ottawa, Ontario, CA
40	OAC_Talbot	II	
41	OAC_Thames		Ottawa, Ontario, CA
42	PRO_280		Ottawa, Ontario, CA
43	RCAT_Angora	II	
44	RCAT_Bobcat		Ottawa, Ontario, CA
45	Savoy	II	
46	Stride	I	
47	SW33_08		Ottawa, Ontario, CA
48	Tiffin	II	
49	Westag_97		Ottawa, Ontario, CA
50	X9063		Ottawa, Ontario, CA

† As per publicly available information, these 50 cultivars are from maturity zones where iron deficiency chlorosis is present.

Table S4. Confusion matrix for a binary classification problem†

	Predicted Positive (Class 1)	Predicted Negative (Class 2)
Actual Positive (Class 1)	True Positive (TP)	False Negative (FN)
Actual Negative (Class 2)	False Positive (FP)	True Negative (TN)

† Three measures of accuracy, sensitivity and specificity of the classifier that were computed from the confusion matrix included:

a) Overall accuracy, which quantifies the fraction of the training dataset that is correctly predicted.

$$Overall\ accuracy = \frac{TP+TN}{TP+TN+FP+FN} \times 100 \quad (4)$$

b) Per-class accuracy is a more refined metric that calculates how the classifier performs for each of the classes. This is useful when the instances in each class vary a lot (i.e. when the classes are *imbalanced*) because accuracy is overestimated due to the class with the most instances dominating the accuracy statistic.

$$Per - class\ accuracy\ = \frac{i-th\ observation\ of\ row\ i}{Sum\ of\ observations\ of\ row\ i} \quad (5)$$

i = 1,…,n, where n = number of classes.

c) Average per-class accuracy is the mean per-class accuracy over these classes.

$$Average\ Per-\ Class\ Accuracy = \frac{1}{n}\sum_{i=1}^{n} Per - class\ accuracy \quad (6)$$

d) The sensitivity and specificity for each class was calculated as true positive rate and true negative rate, respectively:

Sensitivity = TP/(TP + FN) (7)

Specificity = TN/(TN + FP) (8)
